# Coptisine Induces Apoptosis in Human Hepatoma Cells Through Activating 67-kDa Laminin Receptor/cGMP Signaling

**DOI:** 10.3389/fphar.2018.00517

**Published:** 2018-05-18

**Authors:** Li Zhou, Fan Yang, Guobing Li, Jingbin Huang, Yali Liu, Qian Zhang, Qin Tang, Changpeng Hu, Rong Zhang

**Affiliations:** ^1^Department of Pharmacy, The Second Affiliated Hospital, Third Military Medical University, Chongqing, China; ^2^Department of Orthopaedic, General Hospital of Tibetan Military Command Lhasa, Lhasa, China

**Keywords:** apoptosis, cGMP, coptisine, 67LR, hepatocellular carcinoma

## Abstract

Hepatocellular carcinoma (HCC) is the most common primary cancer of the liver. Hence, new anti-liver cancer treatment strategies need to be urgently developed. Coptisine is a natural alkaloid extracted from rhizoma coptidis which exhibits anticancer activity in various preclinical models, including liver cancer. However, the molecular mechanisms underlying the anti-liver cancer effects of coptisine remains unclear. We used flow cytometry to assess the binding of coptisine to 67LR expressed on the surface of SMMC7721, HepG2, LO2 and H9 cells. Then SMMC7721, HepG2 and BEL7402 cells, belonging to the HCC cell lines, were treated with coptisine. The cell viability was detected using a cell counting kit-8 assay. Apoptosis was evaluated using flow cytometry and transferase-mediated dUTP nick-end labeling (TUNEL) assay. Apoptotic-related proteins and tumor death receptor 67-kDa laminin receptor (67LR) were detected using Western blot analysis. The cyclic guanosine 3′,5′-monophosphate (cGMP) concentration was determined using enzyme-linked immunosorbent assay. sh67LR lentivirus, anti67LR antibody, and cGMP inhibitor NS2028 were used to determine how a 67LR/cGMP signaling pathway regulated coptisine-induced apoptosis. Tumor growth inhibited by coptisine was confirmed in a SMMC7721 cell xenograft mouse model. Coptisine selectively exhibited cell viability in human hepatoma cells but not in normal human hepatocyte cell line LO2 cells. Coptisine promoted SMMC7721 and HepG2 cell apoptosis by increasing 67LR activity. Both 67LR antibody and sh67LR treatment blocked coptisine-induced apoptosis and inhibition of cell viability. Coptisine upregulated the expression of cGMP. Moreover, cGMP inhibitor NS2028 significantly decreased coptisine-induced apoptosis and inhibition of cell viability. *In vivo* experiments confirmed that coptisine could significantly suppress the tumor growth and induce apoptosis in SMMC7721 xenografts through a 67LR/cGMP pathway. Coptisine-mediated 67LR activation may be a new therapeutic strategy for treating hepatic malignancy.

## Introduction

Hepatocellular carcinoma (HCC), one of the most common malignancies worldwide, comprises only 10–30% surgical candidates (Gravitz, 2014; Jin et al., 2017). At present, chemotherapy and some oral multikinase inhibitors are primarily used to treat intermediate–advanced HCC (Zhang et al., 2016). However, the treatment benefits are still limited, such as poor curative effects and adverse reactions (Lu et al., 2013; Yoshimoto et al., 2013; Lv et al., 2015). Therefore, more effective pharmacological agents for treating HCC are urgently needed.

Coptisine, an isoquinoline alkaloid, is extracted from rhizoma coptidis. It has a variety of pharmacological effects, such as antibacterial (Kwon et al., 2008), anticachectic (Iizuka et al., 2002; Iizuka, 2016), and lipid-lowering (Kou et al., 2016). Moreover, previous studies demonstrated anticancer activities of coptisine in colorectal cancer, breast cancer, and lung cancer. Huang T. et al. (2017) discovered that coptisine suppressed HCT-116 cell–related tumor growth by promoting tumor cell apoptosis via inhibiting the RAS–ERK pathway. Another study (Rao et al., 2017) found that coptisine could significantly induce mitochondria-mediated apoptosis in non-small-cell lung cancer A549 cells. The present study indicated that coptisine selectively inhibited cell viability in human HCC cell line SMMC7721, HepG2 and BEL7402 cells but not in normal human hepatocyte cell line LO2 cells, suggesting that coptisine might be a potential cancer treatment for HCC. However, its mechanism of action still need further research (Lin et al., 2004; Huang et al., 2015).

Apoptosis is an important mode of programmed cell death. It can be regulated and performed by the activated members of caspase family, such as caspase 3 and 8 (Fan et al., 2017; Huang W. et al., 2017). Moreover, it is well known that caspase activation is initiated and propagated by two major signaling pathways: extrinsic pathway and intrinsic pathway. The extrinsic pathway refers mainly to a death receptor pathway, which is located on the cell surface. It can directly activate caspase 8, and then the downstream caspase 3 is once activated (Li et al., 2015; Boege et al., 2017; Xu et al., 2017).

The 67-kDa laminin receptor (67LR), a new death receptor, is a laminin-binding protein overexpressed in various types of cancer, including multiple myeloma, bile duct carcinoma, colorectal carcinoma, breast carcinoma, and cervical cancer (Song et al., 2012; Kumazoe et al., 2013a; Pesapane et al., 2017). Substantial evidence supported that 67LR regulated the sensitivity and invasiveness of cancer cells to chemotherapeutics. Moreover, recent studies focused more on the effect of 67LR on cancer cell apoptosis (Song et al., 2012; Jovanovic et al., 2015; Zhou Y. et al., 2016). Discovered published study demonstrated that polyphenol (-)-epigallocatechin-3-*O*-gallate (EGCG) could effectively increase apoptosis in myeloma cells after activating 67LR (Dorchies et al., 2009). The underlying mechanism included the cancer-specific cyclic guanosine 3′,5′-monophosphate (cGMP) upregulation by inhibiting the expression of phosphodiesterase 5. The cGMP upregulation could exactly be a rate-determining process of 67LR-dependent cell apoptosis. In addition, Kumazoe et al. (2013a) found that EGCG activated 67LR and then suppressed cancer stem cell properties in pancreatic ductal adenocarcinoma. In normal cells, Activation of 67LR could be an effective agent in suppressing LPS-induced retinal inflammation (Kumazoe et al., 2013a; Huang Y. et al., 2017).

In the present study, it was hypothesized that coptisine induced apoptosis in HCC through a 67LR/cGMP death receptor pathway. Lentivirus knocked-down 67LR were employed to verify the effects of coptisine on apoptosis in SMMC7721 cells *in vivo* and *in vitro*, and further investigated the underlying molecular mechanism downstream of 67LR. These findings provided a novel mechanistic basis for coptisine in HCC treatment.

## Materials and Methods

### Reagents

Coptisine (purity > 98.0% by high-performance liquid chromatography) was provided by Must Biological Technology Co., Ltd. (Chengdu, China) and solubilized in dimethylsulfoxide (DMSO). The cyclic GMP complete enzyme-linked immunosorbent assay (ELISA) kit was purchased from Abcam (Burlingame, CA, United States). The NS2028 reagent was obtained from Beyotime Biotechnology (Jiangsu, China). Allophycocyanin(APC)–Annexin V products were obtained from BD Pharmingen (San Jose, CA, United States). Antibody against 67LR was purchased from Abcam. Antibodies against poly (ADP-ribose) polymerase (PARP), cleaved caspase 3, and cleaved caspase 8 were purchased from Immunoway Biotechnology Company (Jiangsu, China).

### Cell Culture

SMMC7721, HepG2, BEL7402, LO_2_ and H9 cells were provided by the American Type Culture Collection (Manassas, VA, United States). The cells were cultured in Dulbecco’s modified Eagle’s medium (DMEM) supplemented with 10% fetal bovine serum. They were incubated with a humidified atmosphere containing 5% CO_2_ at 37°C.

### Lentiviral Gene Transfer and Gene Silencing

The human sh67LR (5′-GATCCGCCTTCACTAACCAGATCCATTCAAGAGATGGATCTGGTTAGTGAAGGTTTTTTG-3′) and control shRNA plasmids were purchased from Genomeditech (Shanghai, China). Plasmids were co-transfected with lentiviral packaging vectors (hU6-MCS-CMV-mcherry-PGK-Puro-WPRE) into 293FT cells by using Lipofectamine 3000 according to the manufacturer’s protocol. Then the supernatant containing lentivirus was collected and used to infect the SMMC7721 cells. Subsequently, the cells were grown in 5 μg/mL puromycin for selecting the stable cell clone (verification results are shown in **Supplementary Figure S1**).

### Cell Viability Assay

The cell viability was tested using a cell counting kit-8 (CCK-8) (Beyotime, Shanghai, China) according to the manufacturer’s protocol to explore the effectiveness of coptisine treatment for SMMC7721, HepG2, BEL7402 cells and LO_2_ cells *in vitro*. The number of cells was measured after coptisine (0, 12.5, 25, 50 and 100 μM) treatment for 24 h. Also, the number of cells was measured after coptisine (50 μM) treatment for 0, 6, 9, 12 and 24 h. The supernatant of each group was discarded. The cells were then incubated in DMEM basic medium containing CCK-8 for another 2 h at 37°C. An automated microplate reader (Thermo Fisher, Waltham, MA, United States) was used to read the optical density (OD) value at 450 nm.

### Apoptosis Analysis

Apoptotic cells were detected by APC-conjugated Annexin V–APC/DAPI staining (BD Pharmingen) according to the manufacturer’s protocol, and then analyzed with a FACSVantage SE Flow Cytometer (BD Biosciences, San Jose, CA, United States). Both early apoptotic (Annexin V-positive and DAPI-negative) and late apoptotic (Annexin V-positive and DAPI-positive) cells were used for determining cell death.

The apoptotic cells were also detected using an *in situ* cell death detection kit (TUNEL technology) (Roche, Mannheim, Germany) following the manufacturer’s instructions. Images were captured using a Leica scanning confocal microscope (TCS SP5, Leica Microsystems).

### Flow Cytometry Analysis

Fluorescein isothiocyanate (FITC) is one of the fluorescein commonly used for marking alkaloids. We labeled Coptisine with FITC by incubating 50 mM of Coptisine with 0.5 mM FITC in a 100 mM NaHCO_3_ buffer solution (PH = 9.0) for 30 min at room temperature in the dark. Thereafter SMMC7721, HepG2, LO2, and H9 cells were incubated with FITC-labeled coptisine for 30 min and analyzed by flow cytometry in a FACS Vantage SE Flow Cytometer instrument. Heat-inactivated FITC-labeled coptisine was used as a negative control and 1 μg/ml PI was used to discriminate live cells. Flow cytometry data were analyzed with the FlowJo software package (Tree Star, Ashland, OR, United States).

### cGMP Assays

cGMP levels in cells treated with coptisine for about 3 h were measured using the Cyclic GMP Complete ELISA Kit (Abcam), following the manufacturer’s instructions and the OD absorbance was read at 405 nm using an automated microplate reader (Thermo Fisher, Waltham, MA, United States).

### Western Blot Analysis

Western blot analysis was performed as previously described (Zhou L. et al., 2016). Briefly, cell samples were collected and lysed in 1× NuPAGE LDS (Lithium dodecyl sulfate) sample buffer (Invitrogen, Carlsbad, CA, United States) to obtain total protein, whose concentrations were measured using a bicinchoninic acid protein assay kit (Beyotime). Then, 30 μg of sample proteins were separated using sodium dodecyl sulfate–polyacrylamide gel electrophoresis gels and transferred onto nitrocellulose membranes. Then, the membranes were blocked with 5% fat-free dry milk in 1× Tris-buffered saline including 0.05% Tween 20 and incubated with primary antibodies. The following primary antibodies were used: anti-PARP (1:500), anti-Cleaved Caspase-3 (1:500), anti-Cleaved Caspase-8 (1:500) form Immunoway Biotechnology Company, JiangSu, China, anti- 67LR (1:1000) from Abcam, United States, and anti-β-actin (1:1000) from Santa Cruz Biotechnologies. After incubating with horseradish peroxidase–conjugated secondary antibodies, protein bands were detected on a bio-imaging system (Bio-Rad, Berkeley, CA, United States). The ImageJ software was used to measure the densitometric values of the bands.

#### Immunohistochemical Analysis

The cells were mounted on the confocal dish (NEST BD-Falcon, Corning, NY, United States). After treatment in groups, the cells were washed three times with phosphate-buffered saline (PBS), fixed with 4% paraformaldehyde for 20 min, permeabilized using 0.1% Triton X-100 for 10 min, and then blocked with 1% bovine serum albumin for 30 min. Next, the cells were subsequently incubated with target antibodies at 4°C overnight. The following primary antibodies were used: anti-Cleaved Caspase-3 (1:500), anti-Cleaved Caspase-8 (1:500) form Immunoway Biotechnology Company, JiangSu, China, and anti- 67LR (1:1000) from Abcam. Followed by a secondary peroxidase–conjugated goat anti-mouse antibody (Molecular Probes, Invitrogen, United States) for 1 h at room temperature. After washing with PBS, the images were captured using a confocal laser scanning microscope (TCS SP5; Leica Microsystems).

### Xenograft Assay

Male nude mice (5 weeks old) were purchased from Vital River Laboratories (Beijing, China) and fed in a pathogen-free room. All the animal studies were performed in accordance with China’s animal welfare legislation for the care and use of animals and approved by the Third Military Medical University Chongqing, China. SMMC7721 cells (2 × 10^6^ cells per mouse) were subcutaneously inoculated into the right hind leg of 40 mice, which were then randomly divided into four groups (*n* = 10 per group). The other 10 mice were inoculated with sh67LR SMMC7721 cells as the fifth group. After tumor inoculation for 5 days, the mice in all five groups received normal saline, coptisine (50 mg/kg) + immunoglobulin G (IgG), coptisine + anti-67LR (20 μg/mL), coptisine + scramble shRNA, and coptisine + sh67LR (tail intravenous injection, five times a week) for about 6 weeks. Tumor growth and body weights were measured every week, and tumor volumes were calculated according to the formula (length × width^2^)/2. Finally, tumor tissues from representative mice in each group were sectioned, embedded in paraffin, and then processed for hematoxylin and eosin (H&E) and immunohistochemical staining and analysis performed as previously described (Yang et al., 2017).

### Statistical Analyses

Data were expressed as means ± standard deviation and analyzed using SPSS13.0 software (SPSS, Chicago, IL, United States). A one-way analysis of variance (ANOVA) with repeated measures was used to evaluate the significance of the effect of different treatments of coptisine on apoptosis. The Student *t*-test and ANOVA were used for comparing two or more groups, respectively. The results were statistically significant at *P* < 0.05.

## Results

### Coptisine Inhibited Cell Viability in Human HCC Cell Lines SMMC7721, HepG2 and BEL7402 Cells but Not in Normal Human Hepatocyte Cell Line LO2 Cells

**FIGURE 1:**
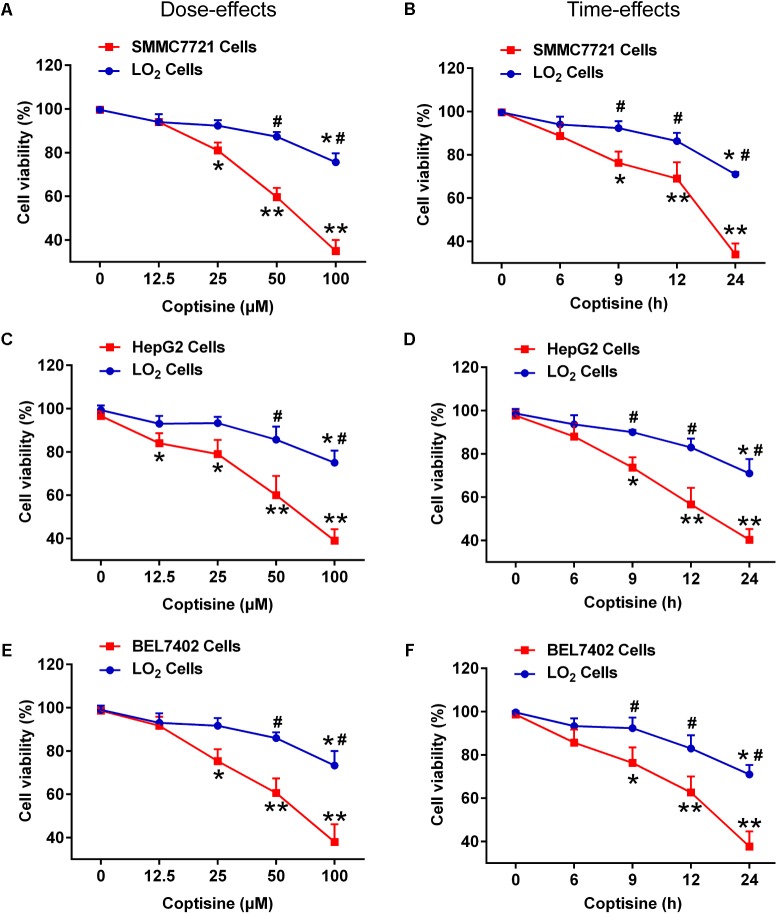
Coptisine inhibited cell viability in SMMC7721, HepG2 and BEL7402 cells but not in LO2 cells. **(A)** SMMC7721 cells, **(C)** HepG2 cells, **(E)** BEL7402 cells and LO2 cells were treated with indicated concentrations of coptisine for 24 h, and cell viability was detected using CCK-8 assay. ^∗^*P* < 0.05 and ^∗∗^*P* < 0.01 compared with the untreated group (control) of the same cells. ^#^*P* < 0.05 compared with the same operated group of SMMC7721 cells. **(B)** SMMC7721 cells, **(D)** HepG2 cells, **(F)** BEL7402 cells and LO2 cells were treated with 50 μM coptisine for 0–24 h, and cell viability was detected using CCK-8 assay. ^∗^*P* < 0.05 and ^∗∗^*P* < 0.01 compared with the 0-h group of the same cells. ^#^*P* < 0.05 compared with the same group of SMMC7721 cells. The results were representative of three independent experiments. Error bars represent mean ± SD.

First, the effects of coptisine on cytotoxicity in human HCC SMMC7721, HepG2, BEL7402 cells and normal human hepatocyte cell line LO2 cells were evaluated using CCK-8 assay. As shown in **Figures 1A,C,E**, exposure of SMMC7721, HepG2 and BEL7402 cells to 12.5 μM coptisine led to a moderate inhibition of cell viability, and 25, 50, and 100 μM coptisine significantly exacerbated this effect. In contrast, some coptisine toxicity was observed in normal human hepatocyte cell line LO2 cells compared with these HCC cells. In addition, a time-effect analysis indicated that 50 μM coptisine could decrease the viability of SMMC7721 cells slightly after 6 h, which became more significant after 9, 12, and 24 h. However, coptisine treatment induced relatively lower toxicity in LO2 cells than in SMMC7721 cells (**Figures 1B,D,F**). These results suggested that coptisine selectively inhibited cell viability in human HCC SMMC7721, HepG2 and BEL7402 cells but not in normal human hepatocyte cell line LO2 cells.

### Coptisine-Induced Apoptosis in SMMC7721 and HepG2 Cells

**FIGURE 2:**
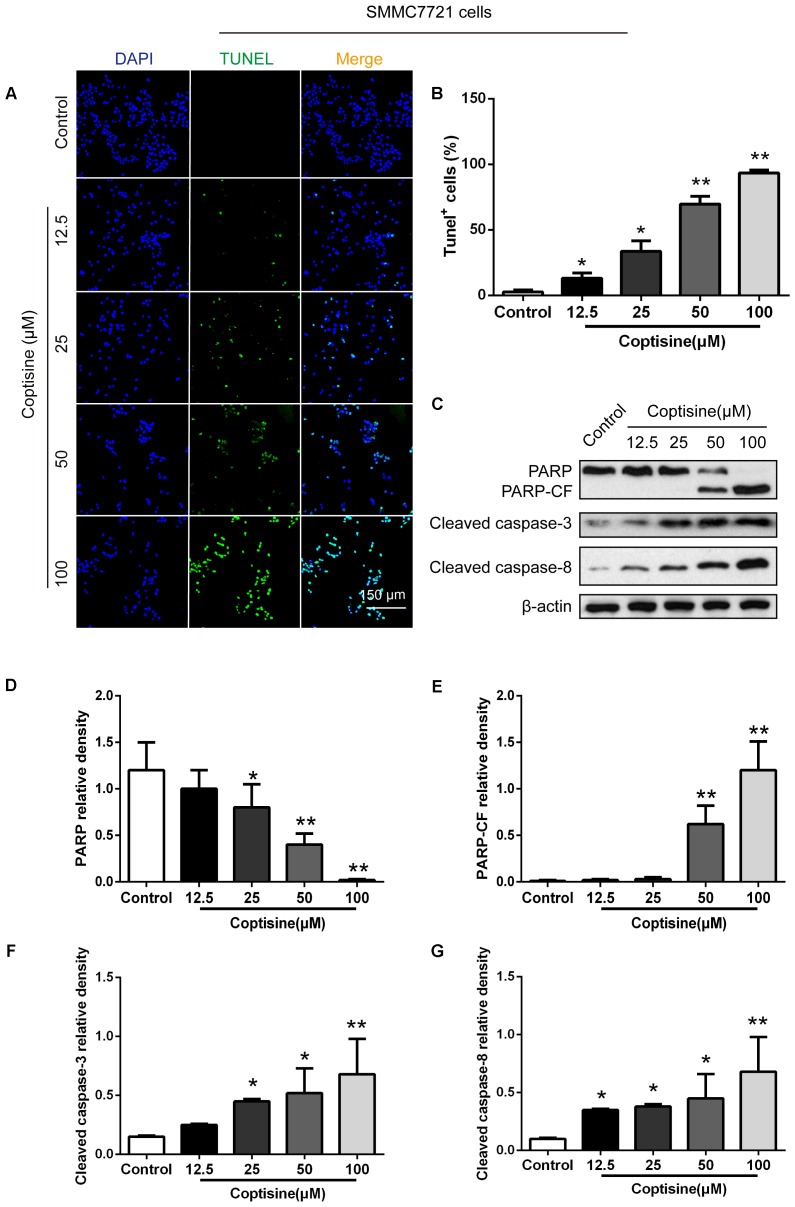
Coptisine concentration-dependent induced apoptosis in SMMC7721 cells. **(A)** The cells were treated with indicated concentrations of coptisine for 24 h. The morphologic changes in apoptotic cells were evaluated using TUNEL staining. Scale bar represents 150 μm. **(B)** The percentages of TUNEL-positive cells were calculated using ImageJ software. **(C)** Total protein lysates were collected after coptisine treatment, and the expression of proteins was detected using Western blotting analysis with the indicated antibodies. PARP-CF means the C-terminal catalytic fragment of PARP. **(D–G)** Relative densities of proteins were analyzed using ImageJ software. The results were representative of three independent experiments. Error bars represent the mean ± SD. ^∗^*P* < 0.05 and ^∗∗^*P* < 0.01 compared with control.

**FIGURE 3:**
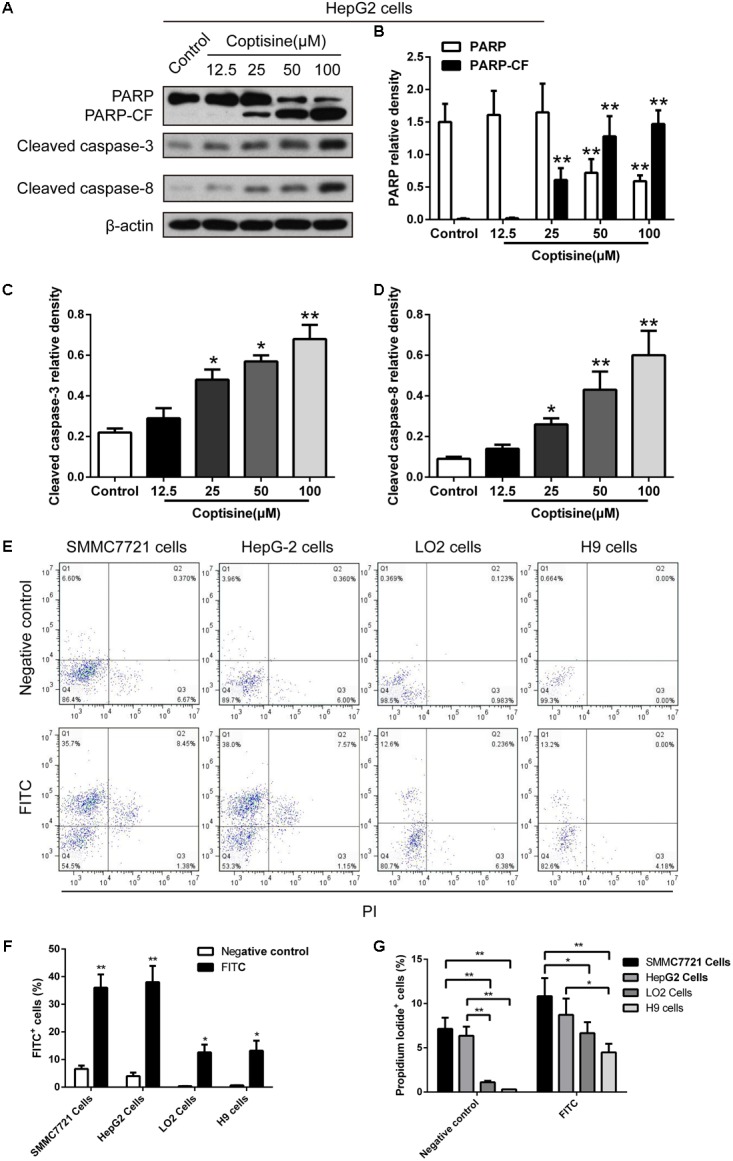
Coptisine concentration-dependent induced apoptosis in HepG2 cells and Coptisine could bind to the surface of hepatoma carcinoma cells. **(A)** After HepG2 cells were treated with indicated concentrations of coptisine for 24 h, total protein lysates were collected, and the expression of proteins was detected using Western blotting analysis with the indicated antibodies. PARP-CF means the C-terminal catalytic fragment of PARP. **(B–D)** Relative densities of proteins were analyzed using ImageJ software. The results were representative of three independent experiments. Error bars represent the mean ± SD. ^∗^*P* < 0.05 and ^∗∗^*P* < 0.01 compared with control. **(E)** 50 mM Coptisine was labeled by 0.5 mM FITC for 30 min (FITC-labeled coptisine), and then added to SMMC7721 cells, HepG2 cells, LO2 cells and H9 cells respectively. 1 μg/ml PI was used to determine living cells. FITC-labeled coptisine was heat-inactivated as a negative control. Cells were filtrated by flow cytometry. **(F)** The percentages of FITC positive cells were counted using FlowJo software (*n* = 3). **(G)** The percentages of PI positive cells were counted (*n* = 3). ^∗^*P* < 0.05 and ^∗∗^*P* < 0.01 compared with the appointed group.

TUNEL technology was used to study coptisine-induced apoptosis so as to explore the effect of coptisine on SMMC7721 cells. Coptisine was shown to significantly increase apoptosis of SMMC7721 cells in a dose-dependent manner (**Figures 2A,B**). Additionally, the expression levels of apoptotic -related proteins, including PARP and cleaved caspases 3 and 8 in SMMC7721 cells (**Figures 2C–G**) and HepG2 cells (**Figures 3A–D**) were detected by Western blot analysis. It showed that the protein expression of C-terminal catalytic fragment of PARP (PARP-CF) and cleaved caspases 3 and 8 significantly increased with the increasing dose of coptisine. Next, to assess if coptisine directly interacts with cell surface expressed 67LR, we performed flow cytometry analysis using FITC-labeled Coptisine. As shown in **Figures 3E–G**, Coptisine effectively binds 67LR expressed on the surface of various cell types including SMMC7721, HepG2, LO2, and H9 cells with apparent higher 67LR expression in malignant cells than in normal cells.

### Coptisine Promoted SMMC7721 and HepG2 Cells Apoptosis by Increasing 67LR Activity

**FIGURE 4:**
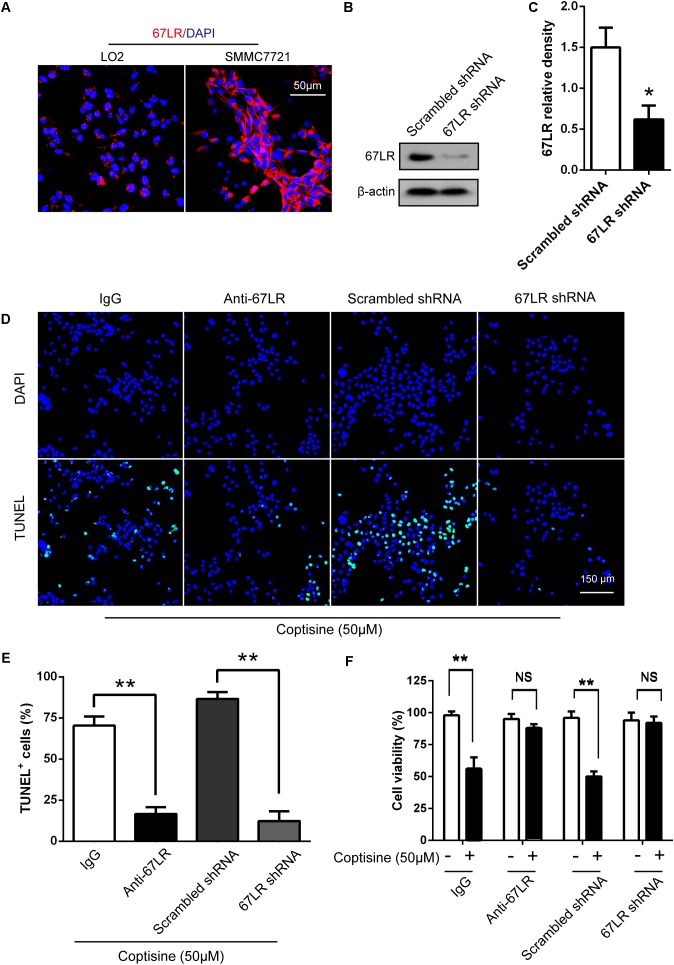
Coptisine promoted SMMC7721 cell apoptosis by increasing 67LR activity. **(A)** LO2 cells and SMMC7721 cells were separately collected and stained with anti-67LR (red) and DAPI (blue) to identify the expression of 67LR using immunofluorescence. The scale bar represents 50 μm. **(B,C)** The expression of 67LR protein was tested in scramble shRNA SMMC7721 and sh67LR SMMC7721 cells using Western blot analysis. The relative density of 67LR expression was analyzed using ImageJ software. ^∗^*P* < 0.05 compared with the scramble shRNA group. **(D,E)** Anti-67LR and sh67LR models were used to detect cell apoptosis after coptisine (50 μM) treatment. Then, morphologic changes in apoptotic cells were evaluated using TUNEL staining. Scale bar represents 150 μm. The percentages of TUNEL-positive cells were calculated using ImageJ software. ^∗∗^*P* < 0.01. **(F)** Cell viability was tested using CCK-8 assay. ^∗∗^*P* < 0.01; NS means no statistical significance. The results were representative of three independent experiments. All data are represented as mean ± SD.

**FIGURE 5:**
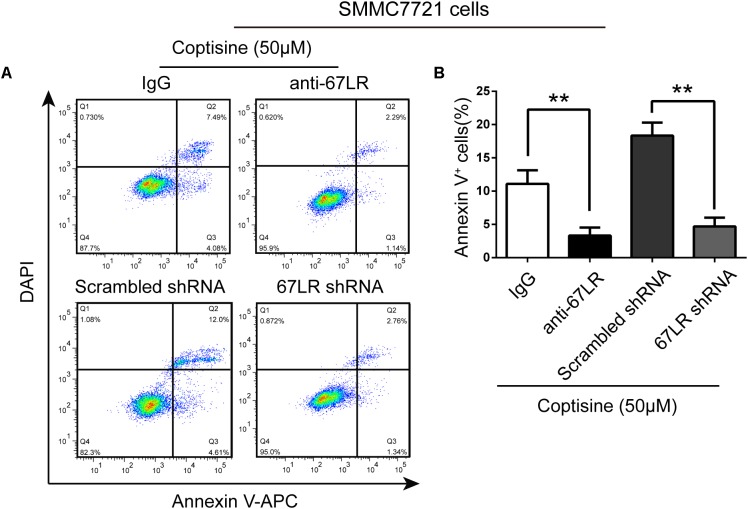
67LR activity significantly influenced the effect of coptisine on SMMC7721 cell apoptosis. **(A,B)** Apoptotic cells were quantified using flow cytometry after staining with Annexin V–APC and DAPI according to the indicated groups. Also, the percentages of apoptotic cells were counted. ^∗∗^*P* < 0.01 compared with the appointed group. Values represent mean ± SD with three replicates.

**FIGURE 6:**
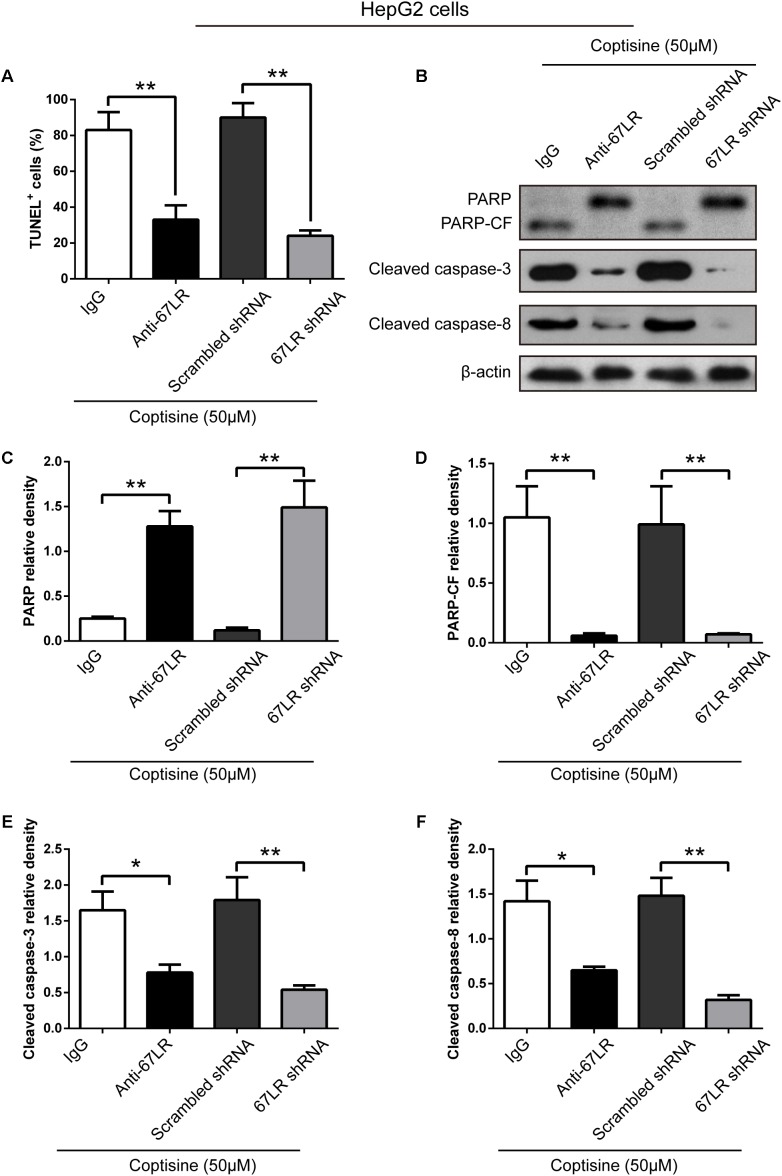
Coptisine promoted HepG2 cell apoptosis by increasing 67LR activity. **(A)** Anti-67LR and sh67LR models were used to detect cell apoptosis after coptisine (50 μM) treatment. Then, morphologic changes in apoptotic cells were evaluated using TUNEL staining. The percentages of TUNEL-positive cells were calculated using ImageJ software. ^∗∗^*P* < 0.01. **(B)** Total protein lysates were collected after treatment, then the expression of proteins was tested by using Western blotting analysis with the indicated antibodies. PARP-CF means the C-terminal catalytic fragment of PARP. **(C–F)** Relative densities of proteins were analyzed using ImageJ software. The results were representative of three independent experiments. Error bars represent the mean ± SD. ^∗^*P* < 0.05 and ^∗∗^*P* < 0.01 compared with the appointed group.

The immunofluorescence results revealed that the expression of 67LR in SMMC7721 cells was much higher than that in human hepatocyte cell line LO2 cells, which was consistent with the finding of other studies that 67LR was highly expressed in cancer cells. Kumazoe et al. (2013b) discovered that a 67LR-mediated pathway contributed significantly to cancer-selective apoptosis. In this study, it was suspected that 67LR was related to the development of HCC induced by SMMC7721 cells, and might be an important target for coptisine-induced apoptosis in SMMC7721 cells. Our immunofluorescence results indicated that the expression of 67LR protein in SMMC7721 cells was significantly higher than that of LO2 cells (**Figure 4A**). A lentiviral vector for sh67LR (**Supplementary Figure S1**) was successfully constructed, and the protein expression of 67LR in SMMC7721 cells was verified after sh67LR lentivirus treatment (**Figures 4B,C**). Then the effects of 67LR knockdown and 67LR antibody on cell viability and apoptosis were examined after coptisine treatment. As shown in **Figures 4D,E, 5A,B**, both TUNEL assay and flow cytometry revealed that coptisine-induced SMMC7721 cell apoptosis decreased after being given 67LR antibody or sh67LR. The results of CCK-8 assay also revealed that both 67LR antibody and sh67LR treatment blocked coptisine-induced inhibition of cell viability (**Figure 4F**). Moreover, similar results were confirmed in HepG2 cells by TUNEL assay (**Figure 6A**) and western blot analysis (**Figures 6B–F**). All the results indicated that coptisine induced SMMC7721 and HepG2 cells apoptosis through a 67LR-dependent pathway.

### Coptisine Induced the cGMP Upregulation After Activating 67LR in SMMC7721 Cells

**FIGURE 7:**
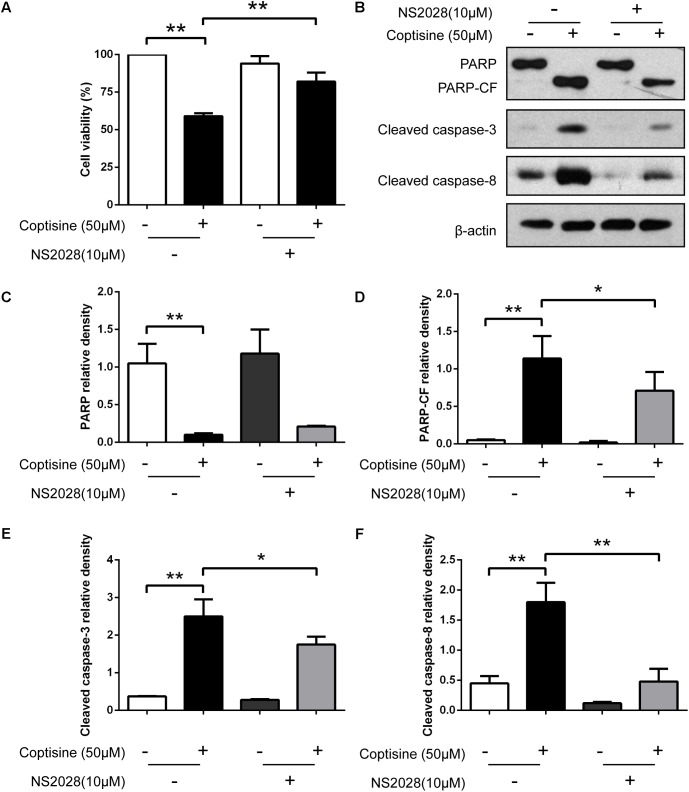
Inhibiting the expression of cGMP could obviously reduce coptisine-induced apoptosis. **(A)** The viability of SMMC7721 cells was detected using CCK-8 assay according to the indicated groups. NS2028 (5 μM) was used to inhibit the expression of cGMP. **(B)** Total protein lysates were collected after coptisine and (or) NS2028 treatment, and then the expression of proteins was detected using Western blot analysis using the indicated antibodies. PARP-CF means the C-terminal catalytic fragment of PARP. **(C–F)** The relative densities of these proteins were analyzed using ImageJ software. ^∗^*P* < 0.05 and ^∗∗^*P* < 0.01 compared with the appointed group (*n* = 3 per group for all the studies). All data are expressed as mean ± SD.

**FIGURE 8:**
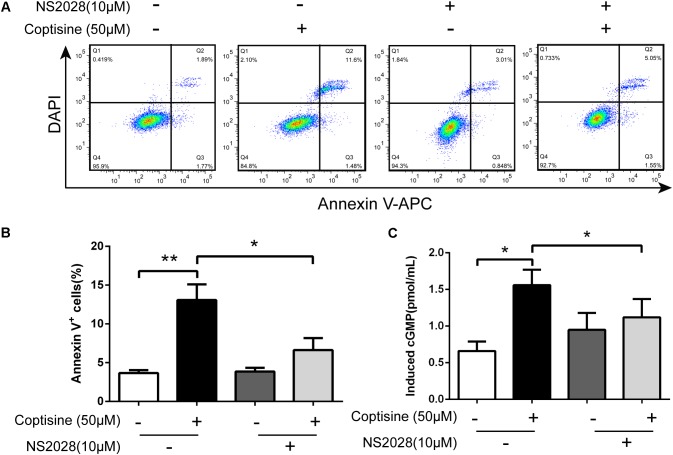
Coptisine induced cGMP upregulation after activating 67LR in SMMC7721 cells. **(A,B)** Apoptotic cells were quantified using flow cytometry after staining with Annexin V–APC and DAPI according to the indicated groups. Also, the percentages of apoptotic cells were counted (*n* = 3). **(C)** The concentration of induced cGMP was measured using an ELISA kit after coptisine and (or) NS2028 treatment (*n* = 6). ^∗^*P* < 0.05 and ^∗∗^*P* < 0.01 compared with the appointed group. All data are expressed as mean ± SD.

67LR acted as a death receptor through cancer-specific cGMP upregulation. And the cGMP upregulation could exactly be a rate-determining process of 67LR-dependent cell apoptosis. Furthermore, NS2028, a high-efficiency and high-specificity inhibitor of soluble guanylate cyclase, was confirmed to directly prevent the cGMP upregulation induced by 67LR agonist (Kumazoe et al., 2013a). The present study investigated, using NS2028 as the inhibitor, whether cGMP was involved in coptisine-induced apoptosis and inhibition of cell viability in SMMC7721 cells. The concentration of cGMP was measured after treatment with coptisine alone and then with a combination of coptisine with NS2028 by using an ELISA kit. Coptisine significantly enhanced the production of cGMP, which could be blocked by NS2028 treatment (**Figure 8C**). Low cytotoxicity was observed after NS2028 treatment alone, and NS2028 significantly reduced coptisine-induced inhibition of cell viability (**Figure 7A**). Western blot analysis revealed that NS2028 observably decreased coptisine-induced PARP cleavage and the expression of cleaved caspases 3 and 8 (**Figures 7B–F**). Moreover, flow cytometry results also showed that NS2028 markedly decreased the number of coptisine-induced apoptotic cells (**Figures 8A,B**). These results indicated that cGMP was involved in coptisine-induced apoptosis and inhibition of cell viability.

### Coptisine Inhibited Tumor Growth, Induced Apoptosis, and Activated 67LR/cGMP Signaling in an SMMC7721 Xenograft Model

The cells were injected subcutaneously into male nude mice to evaluate the *in vivo* activity of coptisine on SMMC7721 and sh67LR SMMC7721 cells. After the appearance of palpable tumors, the mice were divided into five groups randomly: control, coptisine + IgG, coptisine + anti-67LR, coptisine + scramble shRNA, and coptisine + sh67LR. Then, the mice were injected intraperitoneally with normal saline, 50 mg/kg coptisine, and/or 20 μg/mL anti-67LR every day.

**FIGURE 9:**
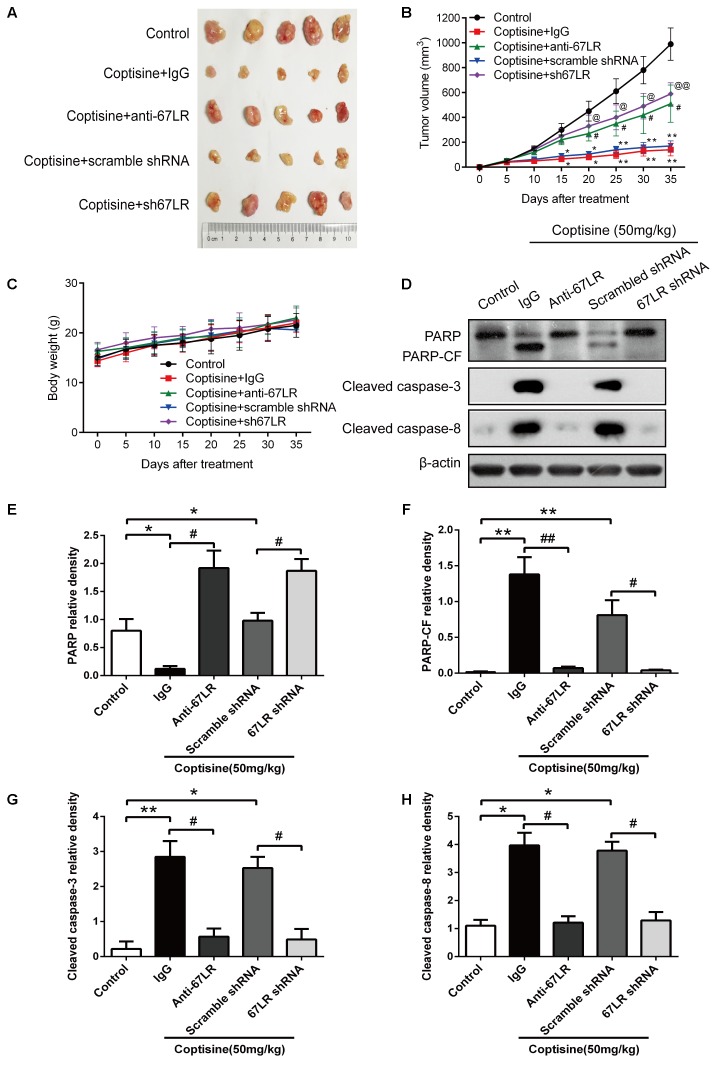
Coptisine inhibited tumor growth and induced apoptosis in a SMMC7721 xenograft animal model. A total of 50 nude mice were inoculated with SMMC7721 cells and then randomly divided into 5 groups (*n* = 10). **(A)** Images were exhibited for five representative tumors from each group after 35 days of treatment. **(B)** Tumor volumes were measured according to the indicated intervals. Data are expressed as mean ± SD. ^∗^*P* < 0.05 and ^∗∗^*P* < 0.01 compared with the same-day results of the control group. ^#^*P* < 0.05 compared with the same-day results of the coptisine + IgG group. ^@^*P* < 0.05 and ^@@^*P* < 0.01 compared with the same-day results of the coptisine + scramble shRNA group. **(C)** Changes in the body weight of mice were recorded during the 35 days of treatment. **(D–H)** Tumors from all the five groups were lysed and collected to detect the expression of objective proteins using Western blot analysis. PARP-CF means the C-terminal catalytic fragment of PARP. The relative densities of these proteins were analyzed using ImageJ software. ^∗^*P* < 0.05, ^∗∗^*P* < 0.01, and ^#^*P* < 0.05 (*n* = 3 per group for all the studies. All data are expressed as mean ± SD.

**FIGURE 10:**
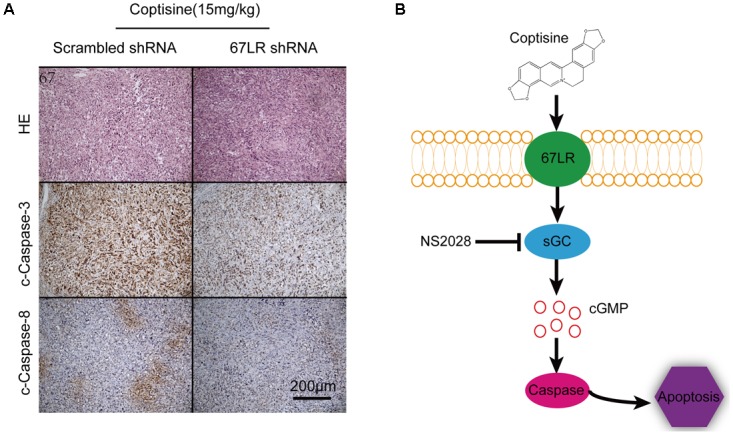
Coptisine induced apoptosis through a 67LR-dependent pathway in a SMMC7721 xenograft animal model. **(A)** Tumors from the coptisine + scramble shRNA and coptisine + sh67LR groups were fixed and stained with H&E to inspect tumor cell morphology. Also, immunohistochemical analysis was used to evaluate the levels of apoptotic -related proteins. Scale bar represents 200 μm. **(B)** An illustration of the molecular mechanism of coptisine-induced apoptosis. Coptisine induced 67LR functional activation and promoted sGC conversion to generate cGMP, resulting in cGMP upregulation, then caspase activation, and finally apoptosis.

As shown in **Figures 9A,B**, the tumor growth was significantly suppressed 15 days after initiating coptisine treatment. These events became more apparent after 25 days of drug exposure. In addition, tumor volumes of the coptisine + sh67LR group were markedly higher than those of the coptisine + scramble shRNA group. Moreover, treatment with anti-67LR antibody significantly blocked coptisine-induced inhibition of tumor growth. However, no statistically significant changes in body weight were observed compared with each group (**Figure 9C**). The mice in the four coptisine treatment groups did not show any other signs of toxicity, such as agitation, indigestion or diarrhea, impaired movement or posture, and areas of redness or swelling. Western blot analysis was used to test the expression of apoptotic-related proteins, including PARP and cleaved caspases 8 and 3, so as to verify that 67LR was involved in coptisine-induced SMMC7721 cell apoptosis. As shown in **Figures 9D–H**, the expression of C-terminal catalytic fragment of PARP and cleaved caspases 3 and 8 was significantly induced in the coptisine + IgG group, whereas treatment with coptisine + anti-67LR and coptisine + sh67LR resulted in an obvious decrease in the expression of these apoptotic -related proteins. Moreover, tumor samples in the coptisine + scramble shRNA and coptisine + sh67LR groups were excised, sectioned, and then analyzed using H&E staining and immunohistochemical analysis. As shown in **Figure 10A**, the results of H&E staining showed that tumors in both coptisine + sh67LR and coptisine + scramble shRNA groups had no typical pathological appearance but inconspicuous inflammation, signs of necrosis, and fibrosis. The immunohistochemical analysis further revealed that mice treated with coptisine and sh67LR displayed decreased immunoreactivity for cleaved caspases 8 and 3, indicating that sh67LR blocked coptisine-induced cell apoptosis in the SMMC7721 xenograft model (**Figure 10A**). Above all, these findings indicated that coptisine significantly inhibited SMMC7721 xenograft growth without obvious side effects, and coptisine-induced cell apoptosis was associated with 67LR activation.

## Discussion

Recent studies showed that some isoquinoline alkaloids and their N-oxides exhibited different pharmacological activities because of a potential structure–activity relationship (Iizuka et al., 2002; Yu et al., 2014). Coptisine, one of the isoquinoline alkaloids, has been reported to exhibit an anti-tumor effect on colon and breast cancer, but little is known regarding its effect on liver cancer (Zhang et al., 2014; Li et al., 2017). Tao et al. reported that the anti-tumor property of coptisine was comparable and even exceeded that of the conventional anti-tumor drugs such as cisplatin and adriamycin, which was similar to the inhibition of the growth of tumor xenografts in a mouse model (Huang T. et al., 2017). Our results demonstrated that coptisine selectively induced apoptosis in SMMC7721, HepG2 and BEL7402 cells (HCC cell lines) and inhibited the tumor growth in xenografts without obvious toxicity, suggesting that coptisine could be developed into a novel anti-tumor agent for treating liver cancer.

The present study, using a variety of techniques and different perspectives, demonstrated that coptisine significantly inhibited cell viability and induced apoptosis in HCC cells but not in LO2 cells. On the one hand, the CCK-8 assay was used to confirm that coptisine significantly decreased cell viability under the influence of mitochondrial dehydrogenase; the number of generated formazan was proportional to the number of living cells. On the other hand, the TUNEL technology was used to verify coptisine-induced cell apoptosis in a concentration-dependent manner through DNA damage. Annexin V–APC/DAPI staining intuitively distinguished the apoptotic cells by labeling them with fluorescent probes. Additionally, the detection of apoptotic-related proteins, including PARP and caspases 8 and 3, by Western blot analysis suggested that caspase-dependent apoptotic pathways were triggered in coptisine-treated HCC cells. Above all, coptisine could induce apoptosis in a large number of cells, but the molecular mechanism underlying coptisine-induced apoptosis needed further investigation.

The 67-kDa laminin receptor (67LR) has been used as a cancer-specific death receptor in a caspase-dependent cell death receptor pathway. 67LR, derived from a 37-kDa laminin receptor precursor, is a non-integrin cell surface receptor located in the cell membrane for extracellular matrix binding with high-affinity laminin-1 (Dorchies et al., 2009; Jovanovic et al., 2015). Recent studies revealed that 67LR was overexpressed in neoplastic cells and associated with an increased invasion and metastasis in human solid tumors (Pesapane et al., 2017). Moreover, 67LR is confirmed to be a new promising target for cancer treatment in some cancer cells (Mocanu et al., 2014; Lu et al., 2016). Kumazoe et al. (2013b) reported that when 67LR together with its natural ligand EGCG was activated, it in turn activated a signal transduction pathway, thus inhibiting growth and inducing apoptosis in cancer cells without detrimentally affecting the normal cells (Kumazoe et al., 2013a,b). Therefore, 67LR, as a new cancer-specific death receptor, promoted cell apoptosis.

This study demonstrated the effect of 67LR on coptisine-induced cell apoptosis. Concretely, 67LR itself was highly expressed in SMMC7721 cells rather than in normal LO2 cells. Also, 67LR-neutralizing antibody or sh67LR could significantly reduce the apoptotic effect of coptisine both *in vivo* and *in vitro*, suggesting that 67LR participated in coptisine-induced apoptosis of liver cancer. In addition, the protein expression of caspases 8 and 3 and PARP in the downstream of cell death receptor pathway was totally influenced by 67LR. Taken together, the results of the present study were consistent with the findings of other studies and indicated that 67LR activation contributed to the coptisine-induced apoptosis.

The findings also provided evidence that a 67LR/cGMP signaling pathway was involved in coptisine-induced apoptosis. Cyclic guanosine 3′,5′-monophosphate (cGMP) is generated by guanylate cyclase. It is an omnipresent second messenger responsible for transducing extracellular signaling pathways. It has been confirmed to be involved in regulating various physiological functions such as platelet aggregation, neurotransmission, and vascular smooth muscle regulation (Saravani et al., 2012). Besides, studies reported that cGMP was crucial in cell proliferation, differentiation, and apoptosis (Tsukamoto et al., 2015; Huang Y. et al., 2017). Particularly, cGMP was confirmed to be an important signaling molecule downstream of 67LR. The cGMP upregulation could exactly be a rate-determining process of 67LR-dependent cell apoptosis (Tsukamoto et al., 2012; Kumazoe et al., 2013a; Montuori et al., 2016). NS2028, a high-efficiency and high-specificity inhibitor of soluble guanylate cyclase, was confirmed to directly prevent the cGMP upregulation induced by 67LR agonist (Kumazoe et al., 2013a,b). The study also showed that cGMP was important in coptisine-induced apoptosis. Concretely, the cell viability significantly increased when the cells were treated with both coptisine and NS2028 compared with only coptisine. Also, the expression of apoptotic -related proteins obviously reduced after the cells were exposed to coptisine and NS2028. Even more intuitively, the concentration of cGMP in SMMC7721 cells increased with coptisine treatment, but it was inhibited on exposure to both coptisine and NS2028. Therefore, coptisine induced apoptosis in SMMC7721 cells probably through a 67LR–cGMP–caspase 3 pathway. In addition, coptisine possessed the function of the anti-inflammation and antioxidant stress in non-tumor cells. Wu et al. (2016) found that coptisine suppressed macrophage inflammatory responses by inhibiting phosphorylation c-Jun NH2-terminal kinase (JNK), p38 mitogen-activated protein kinase (MAPK), and phosphoinositide 3-kinase/Akt (PI3K/Akt). However, coptisine against AAPH induced oxidative stress by activating Akt and JNK (Hu et al., 2017). Therefore, coptisine shows different effects on the same signaling pathway in different cells and models, which opportunely shows its multiple biological functions.

We noticed that the expression of 67-LR was higher in cancer cells than in normal hepatic cells, which was also verified by flow cytometry showing that fluorochrome-labeled coptisine binds to cancer cells with higher affinity than to normal cells. Flow cytometry analysis also showed that 67-LR is expressed on the surface of the T-cell lymphoma line H9. This is relevant since another death receptor, CD95, is expressed on the surface of human lymphocytes and the interaction of CD95 with its respective ligand (Fas ligand) results in cell apoptosis, however, since in this study we did not investigate the expression of 67-LR in primary human lymphocytes, it is unknown whether Coptisine induces apoptosis or activation of normal human immune cells.

## Conclusion

This novel study indicated that coptisine effectively induced death receptor–dependent apoptosis in HCC cells and inhibited tumor growth of xenografts by activating 67LR/cGMP signaling. Collectively, the results supported a hypothetical model of coptisine-induced apoptosis in HCC cells (**Figure 10B**). In this model, coptisine induced 67LR activation and led to cGMP upregulation, resulting in caspase8/3 activation and, finally, apoptosis. Further efforts to explore the mechanism underlying coptisine-induce apoptosis through a 67LR/cGMP signaling pathway might guide the efficient treatment of liver cancer and other hepatic malignancies.

## Ethics Statement

The university’s institutional animal care and use committee approved all the animal studies.

## Author Contributions

LZ, FY, GL, YL, and QZ carried out the experiments. LZ, QT, CH, and JH analyzed the data. LZ, FY, and RZ designed the experiments. LZ and RZ wrote the paper.

## Conflict of Interest Statement

The authors declare that the research was conducted in the absence of any commercial or financial relationships that could be construed as a potential conflict of interest.

## Supplementary Material

FIGURE S1 Verification of lentiviral gene transfer. **(A)** The Lentiviral vector carrying sh67LR gene was constructed. **(B)** Red fluorescence (mCherry) expressing sh67LR plasmids successfully expressed in SMMC7721 cells. Scale bar represents 200 μm.
