# Supplementary figures and images for: Coptisine Induces Apoptosis in Human Hepatoma Cells Through Activating 67-kDa Laminin Receptor/cGMP Signaling
Source: Front Pharmacol. 2018 May 18;9:517. doi: 10.3389/fphar.2018.00517 (PMC5968218; doi:10.3389/fphar.2018.00517)

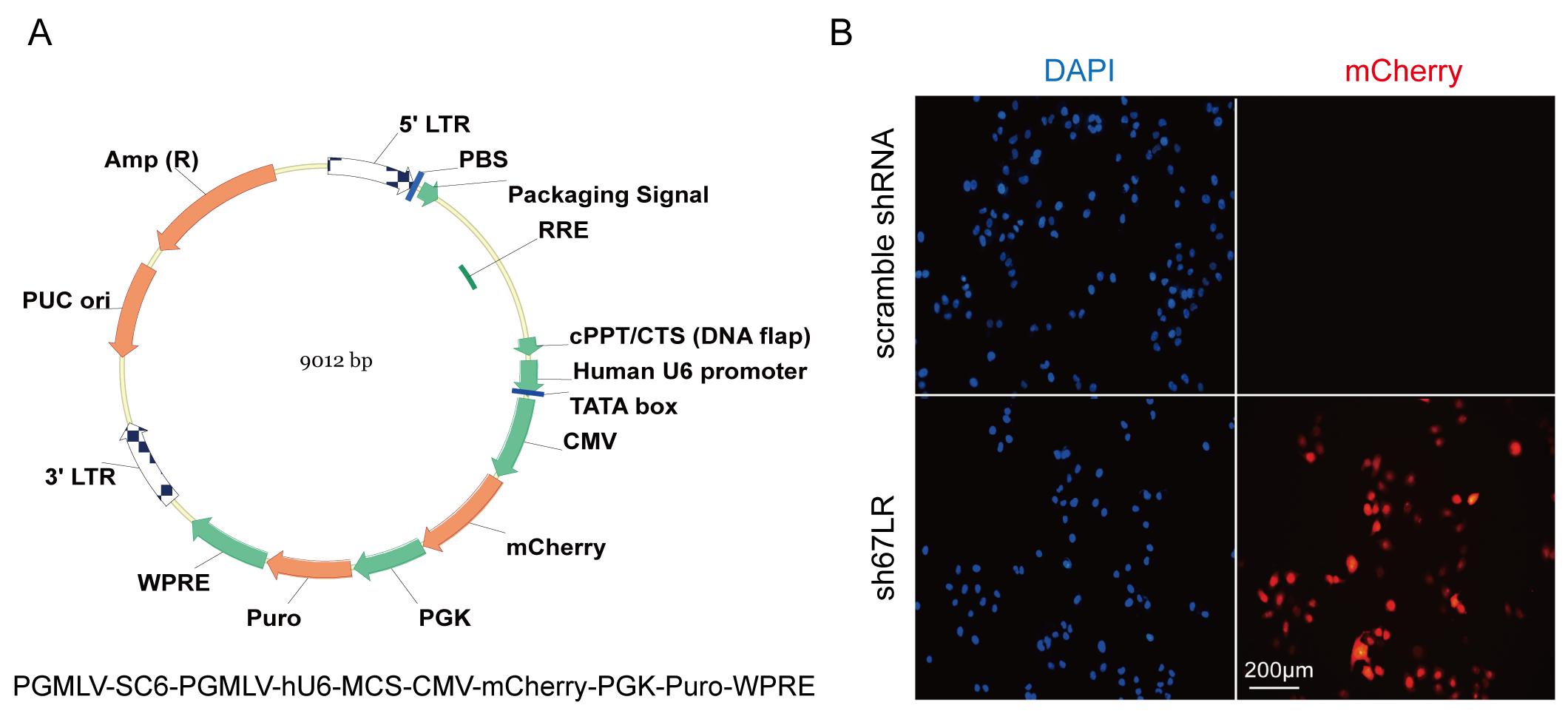

Supplement: FIGURE S1 — Verification of lentiviral gene transfer. (A) The Lentiviral vector carrying sh67LR gene was constructed. (B) Red fluorescence (mCherry) expressing sh67LR plasmids successfully expressed in SMMC7721 cells. Scale bar represents 200 μm. [file Image_1.TIF]
